# 

**Published:** 1890-02-15

**Authors:** 


					L ?Rice ,one penny.
HUE. KJI
Vol. VII.-
-February 15th, 1890.
j-'i * ui. vxi.?xburuary jloov.
ftered at the General Post Office as a Newspaper for
^mission in the United Kingdom and Abroad.
WITH EXT RA ' NURSING SUHVLbMtN 1
Per Aimrm, 6s. ed., post free.
Monthly Parts, 6d.; post free 7?d,
D.tio, per Annum, 7s. 6d., post free.
^er AnHL"mi 6s- e<3-> Post free
* ? '*? "? lmSh . /\ Monthly Parts, 6d.; post free 7?d,
xl

				

